# Development of Lightweight Cricket Pads Using Knitted Flexible Thermoplastic Composites with Improved Impact Protection

**DOI:** 10.3390/ma15238661

**Published:** 2022-12-05

**Authors:** Tauheed Ahmad, Hafsa Jamshaid, Rajesh Kumar Mishra, Vijay Chandan, Shabnam Nazari, Tatiana Alexiou Ivanova, Naseer Ahamad, Sharjeel Ahmed, Michal Petru, Lubos Kučera

**Affiliations:** 1 Protective Textile Research Group, Faculty of Textile Engineering, National Textile University, Faisalabad 37610, Pakistan; 2 Department of Material Science and Manufacturing Technology, Faculty of Engineering, Czech University of Life Sciences Prague, Kamycka 129, 16500 Prague, Czech Republic; 3 Department of Sustainable Technologies, Faculty of Tropical AgriSciences, Czech University of Life Sciences Prague, Kamycka 129, 16500 Prague, Czech Republic; 4 School of Sciences, National Textile University, Faisalabad 37610, Pakistan; 5 School of Materials Science and Engineering, University of Science and Technology of China, Hefei 230026, China; 6 Department of Machine Parts and Mechanism, Faculty of Mechanical Engineering, Technical University of Liberec, 46001 Liberec, Czech Republic; 7 Centre of Transport Technology Components, Faculty of Mechanical engineering, University of Zilina, Univerzitná 8215/1, 010 26 Žilina, Slovakia

**Keywords:** knitted prepreg, composite cricket pad, flax, Kevlar, thermoplastic yarn

## Abstract

Cricket is one of the most popular global sports, and cricket pads are important personal protective gear used for shock absorption and peak deceleration of the impact forces of the cricket ball for both batsmen and wicket keepers. The materials selection of the padding should be considered according to requirements. In the present study, flexible composites were manufactured using knitted unidirectional thermoplastic composite prepregs. Prepregs were fabricated using thermoplastic yarns, e.g., High Density Polyethylene (HDPE), Polypropylene (PP), and Low Melting Polyester (LMPE). Para-aramid (Kevlar) and Flax yarns were used as inlay. The structures were stacked in three and five layers, and hot compression was used to convert thermoplastic yarn into matrix. A total of twelve samples were prepared, and their mechanical properties were evaluated. Tensile and flexural properties, short beam strength, and impact properties were optimized using the multi-criteria decision-making (MCDM) technique for order performance by similarity to ideal solution (TOPSIS). This approach was used to select the best material for use in cricket pads. The candidate samples were ranked using statistical techniques. The optimum sample was found to be FP5, i.e., Flax with polypropylene using five layers, which exhibited the maximum impact strength. The results showed that the mechanical properties were improved in general by increasing the number of layers. The significance and percentage contribution of each factor was obtained by ANOVA (α = 0.10) and pie chart, which showed Factors A and C (inlay yarn and number of layers) to be the main contributors. The optimal samples showed superior impact-related performance compared to a market sample cricket pad.

## 1. Introduction

Cricket is one of the most admired games in the world, with approximately 2.5 billion fans. It is the national game of the UK, and is popular in India, Pakistan, Sri Lanka, Australia, New Zealand, and other countries [1]. Certain equipment is required for the sport, such as the cricket bat, cricket ball, wicket, and protective accessories, e.g., cricket pads, helmet, gloves, and thermoregulating protective gear [2,3]. The market for cricket equipment has expanded over the years with advancements in technology and materials. Personal protective equipment (PPE) has become increasingly important in professional sports. Although cricket is a noncontact sport, impact injuries are very common. In cricket, balls are delivered at very high speeds ranging from 25 to 45 m/s. At such high speeds, the force generated upon impact can be detrimental to player safety. In lieu of such events, it is obvious that continuous and thoughtful improvements must be made to the protective equipment utilized by batsmen [4]. Cricket pads are essential for players during batting and wicket keeping for the safety of the knees and lower legs. Cricket pads need to provide shock absorption and peak deceleration of the impact forces of cricket ball for batsmen and wicket keepers [5,6]. Cricket pads are made up of several materials which have good shock absorption and impact resistance. Pads are used to minimize the impact of a high-speed ball to protect players from injuries during batting and wicket keeping, because in these positions the player must face the cricket ball, which can be dangerous and cause injury to the players [7,8,9,10].

Conventionally, leather has been used to manufacture the outer layer of cricket pads. However, leather-based pads are heavy; in recent years the development of PVC (polyvinyl chloride) and PU (polyurethane) has made it possible to manufacture lightweight pads. PVC is a synthetic polymeric material used as a replacement for leather, as it is cheaper, more durable, and lighter than leather. PVC has high chemical resistance, which makes it difficult to recycle. Because of this, PVC has lost importance in recent years [11,12]. PU is now more widely adopted, as it is more readily recyclable, and is as cheap and durable as PVC. PU can be converted into a high-density foam to be used as a lightweight shock absorber to make lightweight pads. The outer facing of a cricket pad is made up of Polyurethane (PU)/Polyvinyl Chloride (PVC), while different materials are used as padding according to their inherent properties of impact resistance and shock absorption [13]. Traditionally, cane (light wood) in stiff form is used in pads to provide rigidity and protection inside cricket pads. In modern equipment, cane has been replaced by High-Density Foam (HDF), and is now largely found in cheaper and better quality pads. The major benefit of high-density foam is that it is extremely lightweight, as it is largely made up of air. However, such synthetic materials are derived from non-renewable natural resources, e.g., oil, which contributes to global warming and has limited end-of-life disposal routes [11,12]. Research on potential sustainable innovation opportunities is needed. Cricket pads with greater impact protection while focusing on the comfort and safety of players simultaneously are becoming necessary. Natural fibers such as flax, jute, sisal, silk, and coir are inexpensive, with relatively lower density and higher toughness. These fibers are lightweight, renewable, and biodegradable. Studies show that flax fibers have high specific strength and modulus along with low density, which makes it remarkably effective for use in composite materials [13]. For high-end application, aramid fibers, e.g., Kevlar, are preferred due to their high strength, light weight, and shock absorption properties, which make them suitable for use in cricket pads [14].

In recent years, fiber-reinforced composites have been widely used in many engineering sectors owing to their inexpensiveness, light weight, improved strength, and high stiffness. Different types of composites can be prepared depending on the type of reinforcement and matrix materials. The direction of reinforcement is one of the decisive factors governing the mechanical properties of a composite [14,15]. Prepregs are composite reinforcing materials that have been pre-impregnated with a thermoplastic or thermoset resin. While the production procedure is quicker for prefabricated parts, they are cured by hot compression moulding. Prepregs are prepared using the knitted unidirectional reinforcement method, as mentioned in the literature [16].

Development of cricket pad with environmentally friendly materials is becoming necessary, as the world is curbing its fuel consumption and corresponding CO_2_ emissions. Leather, cane, and HDF have been used for developing cricket pads for impact protection; however, these materials all have their own advantages and pitfalls. There are no studies available on the development of cricket pads with flexible composites reinforced by high-performance knitted fabrics and impregnated with thermoplastic resins to improve impact protection. The existing research indicates that there has been limited consideration of the environmental implications and materials used. This investigation is focused on the performance characteristics of a flexible composite for use as protective gear in cricket. Further, the TOPSIS multi-response optimization method was employed. Thus, this study is aimed on one hand towards the use of renewable materials, and on the other hand towards realizing an improvement in impact properties.

## 2. Materials and Methods

### 2.1. Materials

For the preparation of composite prepregs, five different types of materials, namely, Kevlar, Flax, Polypropylene (PP), High Density Polyethylene (HDPE), and Low Melting Polyester (LMP) multifilament yarns, were used. All the yarn materials were imported from China (Senyu group, Anqiu City, China). Kevlar and Flax yarns were used as reinforcing material; both of these yarns have linear densities of 6000 Denier. The other three types of thermoplastic yarn materials, i.e., low melting polyester, high density polyethylene, and polypropylene, were used as matrix in all knitted structures during hot compression. The linear densities of all thermoplastic yarns was 600 Denier. The properties of the materials used in this study are provided in Table 1.

Thermoplastic polymers were selected on the basic of their melting point and their commercial availability in yarn form. Their selection was on the basic of lower melting temperature than the reinforcement, which allows easy and uniform impregnation to be achieved. Thermoplastic matrices such as LMP, PP, and PE are ductile, easy to process, and simple to recycle.

Reinforcing yarns were selected based on superior mechanical performance. While Kevlar is well known for high end engineering applications, flax was selected as it is a natural-origin cellulosic fiber which is an ecofriendly option for reinforcement while exhibiting relatively better mechanical performance compared to other natural fibers.

The HDPE used in this study was in the form of multifilament yarns, which are composed of fibers/filaments with very high orientation of polymeric chains, increasing the crystallinity. This is typical for polymeric fibers; their density is slightly higher than the corresponding polymers.

The PP used in this study was in the form of multifilament yarns. Because the yarns were directly procured from the supplier, there could be plasticizer included. This may be a reason for the high elongation in PP multifilament yarns.

Our objective was to study the nature of composites impregnated by different thermoplastic resins used in yarn form. Thus, the selection of material constituted a relatively lower density and molecular weight PP, in contrast to a relatively higher density and higher molecular weight material, e.g., HDPE or an intermediate material, e.g., low melting polyester filament yarn for comparison purpose.

**Figure 1:**
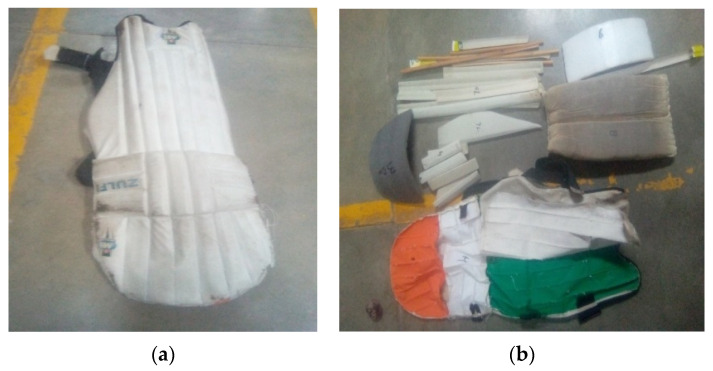
Market sample of cricket pads: (**a**) commercial cricket pad and (**b**) different types of reinforcement in pads.

Cricket pads, as shown in Figure 1, were purchased from the market for comparison purposes.

### 2.2. Methods

**Figure 2:**
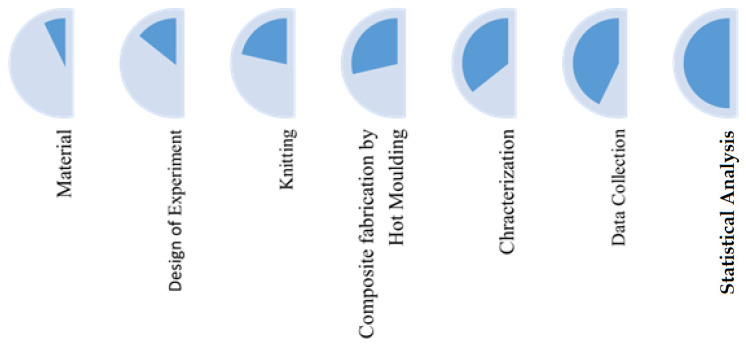
Process flow of this study.

The process flow adopted for this study is shown in Figure 2.

### 2.3. Design of Experiment

Design of Experiment (DOE) is an effective tool for studying the effects of more than one factor on multiple responses. Three different factors, each with three levels, and another two factors with two levels each, were used. The experimental factors and their levels are shown in Table 2, and the design of the experiment is presented in Table 3.

**Figure 3:**
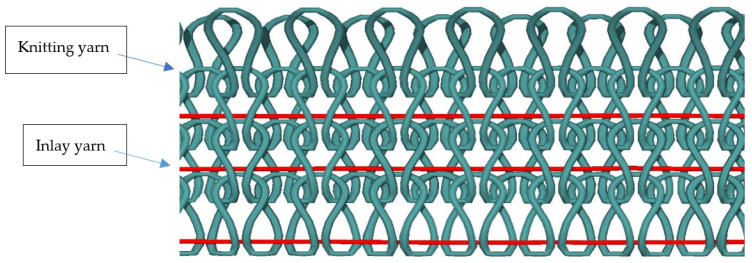
Knitting and inlay yarns.

**Figure 4:**
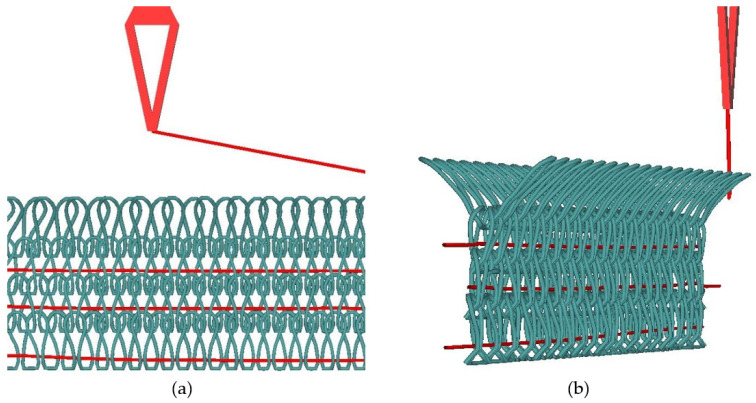
Description of structure on machine: (**a**) front view on machine and (**b**) side view.

Knitting of the above selected samples was carried out on a double bed electronic flatbed knitting machine (Model SES 182 FF) with two cams, eight feeders, and a gauge (E) 7. An inlay structure was used for knitting, as shown in Figure 3 and Figure 4. High Density Polyethylene, Low Melting Polyester, and Polypropylene were used as knitting yarn and Para-Aramid (Kevlar) and Flax (natural cellulosic material) were used as inlay yarn.

### 2.4. Hot Compression Moulding

**Figure 5:**
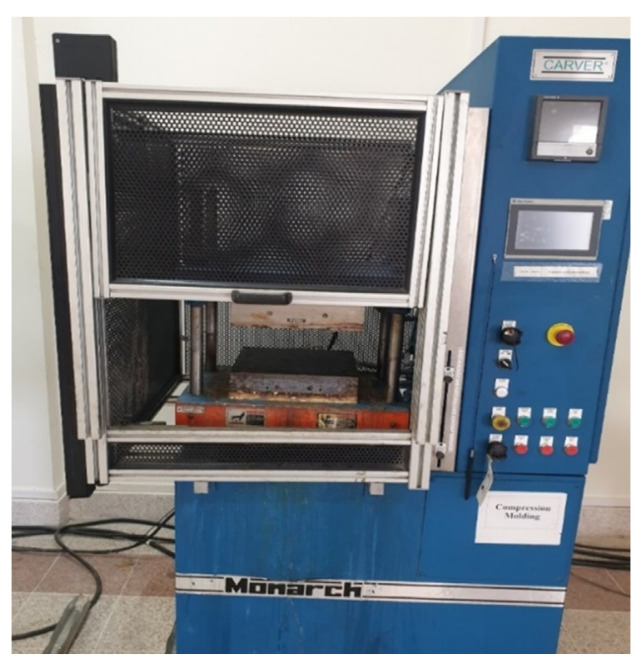
Hot compression moulding machine.

The composite samples were fabricated using two different stacking sequences. Knitted fabric specimens were cut into the required size (30 cm × 30 cm) and placed with stacking sequence of (0,90,0) for three layers and (0,90,0,90,0) for five layers. Then, the specimens were placed in a compression molding machine, as shown in Figure 5, to melt the thermoplastic yarns. A pressure of four tons and temperature of 180 °C were applied for 30 min to achieve the targeted density with homogeneity. The specimens were allowed to cool at 65–75 °C with applied pressure. The composite samples were then removed from the hot compression molding machine.

### 2.5. Characterization of Samples

#### 2.5.1. Physical Parameters of Knitted Fabric Specimens

The physical parameters of all knitted specimens are provided in Table 4.

#### 2.5.2. Mechanical Testing of Composite Samples

**Figure 6:**
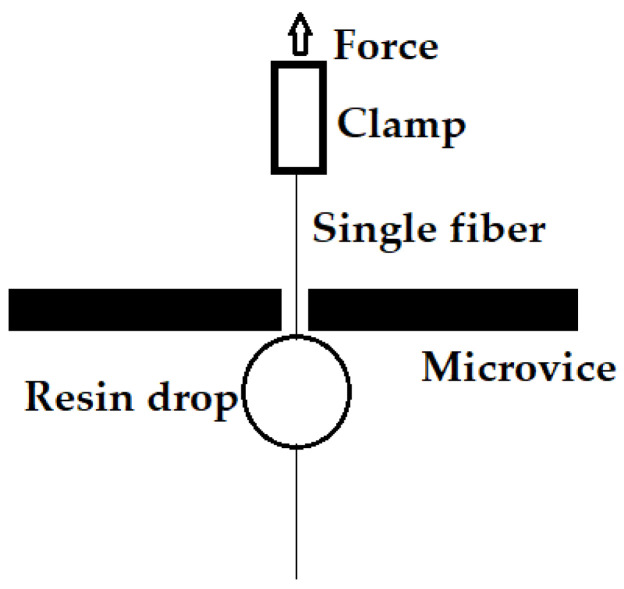
Schematic of single fiber pull-out test.

Fiber pull-out tests were conducted according to the ASTM D7913/D7913M standard [17] in order to determine the adhesion strength between various types of fibers in the fabricated composite materials. The schematic of the single fiber pull-out test is shown in Figure 6.

Tensile strength tests for all composite samples were performed on a UMT Z 100 All-round line (Zwick, Germany) as per the ASTM D3039 standard [18].

The flexural properties of the composite samples were determined according to the ASTM D7264 standard test method using the three-point bending test [19]. The Zwick Universal testing machine was used for the three-point bending test by changing clamps.

A short beam shear strength test of the thermoplastic composites was carried out on the universal testing machine (Zwick/Roell, Brno, Czech Republic) according to the ASTM D2344 testing standard [20]. Short beam strength or inter-laminar shear strength were used to evaluate the fiber–matrix bonding, using a straightforward mode II transverse shear loading test designed to gauge the interfacial bonding quality.

The impact properties of all thermoplastic composite samples were determined according to testing standard ISO 179-2 on a Charpy impact tester [21]. The Charpy test method assesses the material’s toughness or impact strength when it has a defect or notch and is subjected to rapid loading circumstances. Impact strength is the energy, which is absorbed by the material before fracture, while energy absorption is directly related to the brittleness of the material. A specimen size of 10 mm × 100 mm was prepared and placed in the clamps of the pendulum impact tester. The applied force was measured and recorded until fracture of specimen, then impact strength was calculated in KJ/mm^2^.

To compare the results of the impact properties, market samples of cricket pads and developed composite samples were tested using the drop weight method. The Zwick/Roell drop (Zwick/Roell, Brno, Czech Republic) impact tester was used to conduct the impact test in accordance with the ASTM D7136 standard [22].

### 2.6. Microscopic Images and Analysis

Optical images of all tested samples were obtained using an optical microscope (OPTIKA C-B10, Optik, Cech, Luhačovice, Czech Republic) with a magnification of 1.5×. The fracture surfaces were analyzed to determine the failure mode.

### 2.7. Statistical Analysis

To achieve the optimum sample for the cricket pads, the TOPSIS (technique for order performance by similarity to ideal solution) multi-response optimization technique was employed [23]. The TOPSIS method is used to obtain the closest ideal solution which is farthest from the negative ideal solution. This method requires information on the relative importance of properties that should be considered in the selection process. The first quality criterion is impact performance, which shows how much energy absorption flexible pads are capable of, which is one of the most important requirements for cricket pads. The order of preference that is a necessary part of this analytical technique is as follows: impact strength, bending strength, tensile strength, and short beam strength. ANOVA was employed to identify the statistical significance and percentage contribution of each factor with respect to each particular response proposed by TOPSIS. Finally, the market sample cricket pad was compared with the optimum sample obtained through the above analysis.

## 3. Results and Discussion

The average results of our mechanical tests of the developed composite samples were calculated, and are shown in Table 5.

### 3.1. Tensile Properties

**Figure 7:**
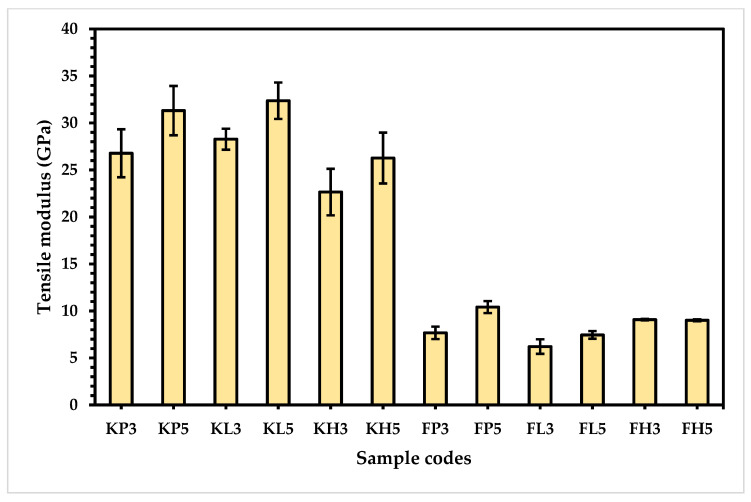
Tensile modulus of developed composite samples.

The tensile properties play a vital role in the characterization of composites prepared for cricket pads. The tensile modulus and tensile strength of the composite samples were investigated and analyzed. The tensile modulus was calculated, and plotted for comparison of all prepared samples, and is presented in Figure 7.

From Table 5 and Figure 7, the results reveal that the Kevlar-based samples possess higher tensile modulus compared to the flax-based composites because of the para-aramid component present in the chemical structure of Kevlar, which is inter-chained via strong linkages in a repeated manner. In addition, hydrogen bonding plays a crucial role in strengthening the Kevlar composites [24]. On the other hand, the natural flax fiber is mostly composed of long-chain cellulose, which is interconnected via ß-1,4 linkages [19]. Interestingly, the degree of polymerization is 18,000, resulting in a more flexible and softer fiber than Kevlar [25]. These results are supported by the fiber adhesion strength, as shown in Table 5.

In addition, the five-layered composites of all categories exhibit a higher tensile modulus than the three-layered composites within the same type of composites, which can be directly attributed to the higher number of layers, which build strong interlaminar bonds between the various layers of polymers and fibers, resulting in a higher tensile modulus. However, in the special case of flax-based high-density polyethylene (HDPE) composite (FH), the five-layered composite FH5 has a slightly lower modulus than the three-layered composite FH3. This could be because of the thicker composite being able to bear the linear, stiff, and highly crystalline structure of HDPE, resulting in lower tensile strength of FH5 compared to FH3 [26].

As for the Kevlar-based composites, the low melting polyester composite (KL) shows a higher tensile modulus (28.27 GPa and 32.36 GPa) than KP (26.77 GPa and 31.31 GPa), followed by KH (22.65 GPa and 26.26 GPa) for both three-layered and five-layered composites, respectively. This might be due to smoother and more uniform rod-like longitudinal micro-fibers, which are oriented and interlinked with each other and possess a higher crystalline structure, resulting in a higher Young’s modulus, tensile strength, and tensile modulus. On the other hand, the low tensile modulus of the high-density polyethylene-based composites (KH) could be caused by the HDPE. When the high-density sample (KH) is exposed to the tensile test conditions, the stretching of the composite produces friction between the interlinked fibers, which can produce heat and melt the polyethylene due to the thermoplastic behaviour of HDPE, resulting in a low tensile modulus [27].

**Figure 8:**
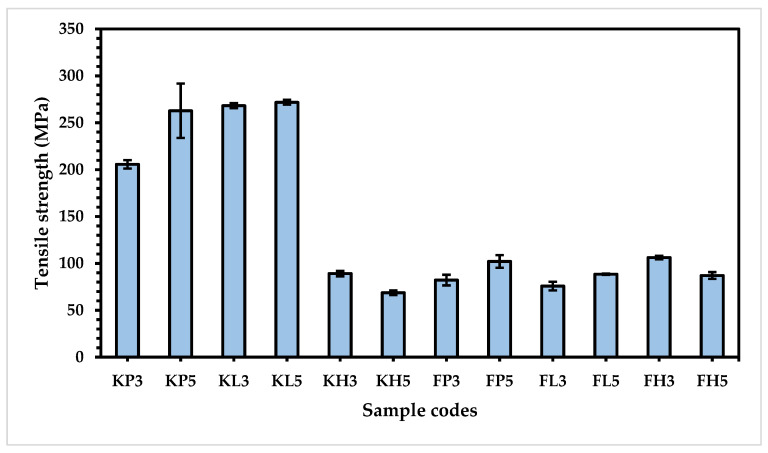
Tensile strength of composite samples.

In comparing the flax-based composites, FP shows the highest tensile modulus (7.67 GPa and 10.41 GPa), followed by FH (9.07 GPa and 9.00 GPa) and FL (6.21 GPa and 7.45 GPa) in three-layered and five-layered composites, respectively. This can be attributed to the highly oriented spherulites in the direction of force, which are converted into microfibrils that create a strong bonding with the flax fibrils, resulting in a high tensile modulus [28]. On the other hand, FH has a higher tensile modulus than FL, which can be assigned to the higher stiffness/rigidity and compression strength of HDPE, as proven by the results presented in Figure 8.

The tensile strength of all prepared composites was investigated as well. The tensile strength of the Kevlar-based samples is higher than the flax-based composites due to their compact and high hydrogen bonding within the interlinked chain and to the aromatic structure of Kevlar. These results are supported by the fiber adhesion strength, as shown in Table 5. Depending on the number of layers for the Kevlar-based composites, obviously the five-layered composites have a higher tensile strength than the three-layered composites due to their strong confinement and interlinking bonding between the layers. Interestingly, the tensile strength of five-layered composites for HDPE (68.68 and 87.08 MPa) is lower than for three-layered composites (89.13 and 106.27 MPa), respectively, in the cases of both the Kevlar and flax-based composites. This can be attributed to the flexible nature of polyethylene. When the number of layers of HDPE increases, the flexibility decreases, leading to lower tensile strength.

The comparison of the three-layered and five-layered KL composites showed a higher tensile strength (268.26 and 271.62 MPa) than the KP composites (205.67 and 262.81 MPa) due to the strong intermolecular forces of low melting polyester and the hydrogen bonding of interlinked and unidirectional aromatic chains in Kevlar [29]. Further analysis reveals that both three-layered and five-layered FP composites show a higher tensile strength (82.18 and 102.10 MPa) than FL composites (75.80 and 88.45 MPa). This could be attributed to the higher tensile strength of polypropylene compared to polyester, as well as to the highly crystalline and geometrically regular (isotactic) structure [30]. Moreover, the lignin, pectin, and hemicellulose present in the flax fibers contribute to strong bonding between flax and polypropylene, as confirmed by the results shown in Figure 8.

### 3.2. Bending Properties

**Figure 9:**
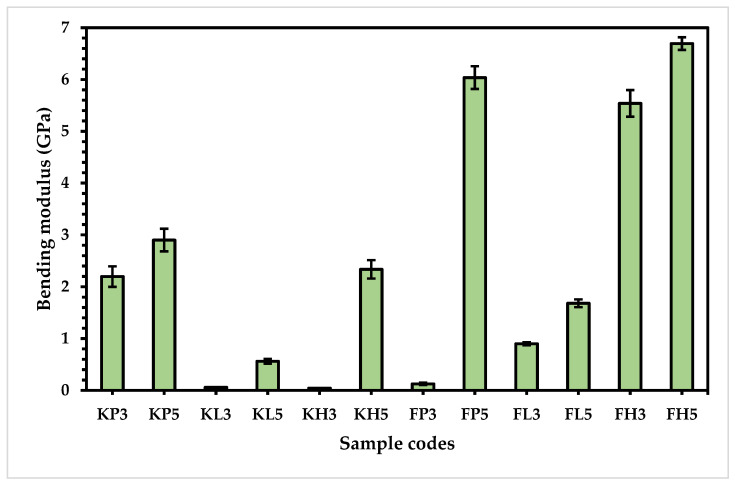
Bending modulus of composite samples.

To analyze the bending modulus (sometimes known as the flexural modulus), all the prepared samples were tested by a flexural tester, with the results plotted in form of the bar chart shown in Figure 9. These results show that the bending modulus of the flax-based composites is higher than the Kevlar-based composites due to better bonding of the rough flax fibers compared to the smoother Kevlar fibers. This might be responsible for a stiffer composite with natural fiber reinforcement [31,32]. These results are supported by the fiber adhesion strength, as shown in Table 5.

Furthermore, the five-layered composites in all samples show a higher bending modulus than the three-layered composites in both the flax and Kevlar-based samples. This is certainly due to the long chains of cellulosic linkage and higher degree of polymerization. The five-layered samples show higher bending modulus than the three-layered samples, as proven by the results shown in Figure 9. For the Kevlar-based composites, both the three-layered and five-layered composites of KP revealed the best bending modulus (2.20 and 2.90 GPa), followed by KL (0.06 and 0.56 GPa) and three-layered KH (0.04 GPa). Interestingly, the bending modulus of five-layered KH (2.34 GPa) is higher than KP3 (2.20 GPa) and lower than KP5 (2.90 GPa), which could be due to the flexible nature of HDPE; in comparison with KP5, KH5 has a lower bending modulus due to its stiffer nature, in spite of having the same number of layers (5) [33,34].

Both the three-layered and five-layered composites of flax and HDPE (FH3 and FH5) show the highest bending modulus (5.54 and 6.69 GPa, respectively) compared to the bending modulus of FL3 (0.90 GPa), FL5 (1.68 GPa), and FP3 (0.12 GPa) due to the more malleable and less branching structure of HDPE, which contributes to better intermolecular bonding between the HDPE and flax fibers, and thus exhibits the highest bending modulus [26]. Interestingly, the bending modulus of FP5 (6.04 GPa) is higher than FH3 (5.54 GPa) and lower than FH5 (6.69 GPa). This may be attributed to the intermediate level of crystallinity between FH3 and FH5 due to the isotactic block in the structure of polypropylene [35]. However, when the number of layers is the same, obviously FH5 exhibits a higher bending modulus than FP5 due to the elastic and flexural nature of HDPE [35].

**Figure 10:**
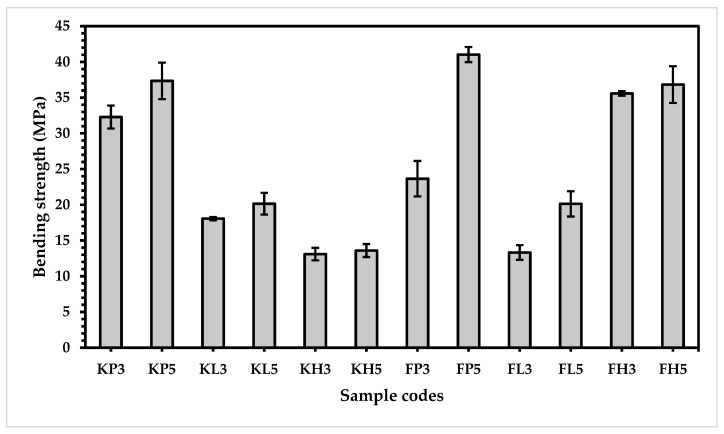
Bending strength of composite samples.

After the evaluation of the bending modulus, the bending strength of all the prepared samples was further investigated. The results explain interesting and unique facts for each composite, as shown in Figure 10. The figure reveals that the bending strength of all five-layered composites of both types of reinforcing materials is higher than the same composites with three-layered structures. It is clear that the five-layered composites in each category have a higher share of elastic and soft polymers such as (polypropylene (P), low melting polyester (L), and high-density polyethylene (H). Due to their long chains of polymers and higher degree of polymerization, the five-layered samples show higher bending strength. It is very crucial to note that flax fiber is softer than Kevlar, resulting in higher bending strength of the flax-based samples [36].

Moreover, the KP composites demonstrate the highest bending strength (13.10 and 37.35 MPa), as compared with the KL composites (18.06 and 20.15 MPa) and followed by the KH composites (13.10 and 13.59 MPa) for three-layered and five-layered samples, respectively. The credit for this highest bending strength is due to polypropylene, which has a linear structure based on C_n_H_2n_ as well as a highly crystalline and geometrically regular structure (isotactic) compared to low melting polyester and high-density polyethylene. Interestingly, high-density polyethylene exhibits a lower bending strength than low melting polyester, which is because of its stiffer and more crystalline and rigid structure [37].

For the flax-based samples, the FP composite exhibits the highest bending strength (23.65 and 41.03 MPa) compared to the FL composites (13.32 and 20.13 MPa) and FH composites (35.58 and 36.82 MPa) for three-layered and five-layered samples, respectively, which is due to the combined effect of flax (a very crystalline polymer system and extremely long polymer with more hydrogen bonds) and polypropylene (a highly oriented spherulite in the direction of force) [38,39,40]. It is worth noting that the FL composites (13.32 and 20.13 MPa) show lower bending strength than the FH composites (35.58 and 36.82 MPa) for both three-layered and five-layered samples. Although polyester is very elastic in nature and yields high crystallinity and great molecular orientation compared to HDPE, it has lower elongation than HDPE, resulting in lower bending strength [35].

### 3.3. Short Beam Strength

As these composites are made for cricket pads, it is very crucial to measure the short beam strength and impact strength of all prepared composites.

**Figure 11:**
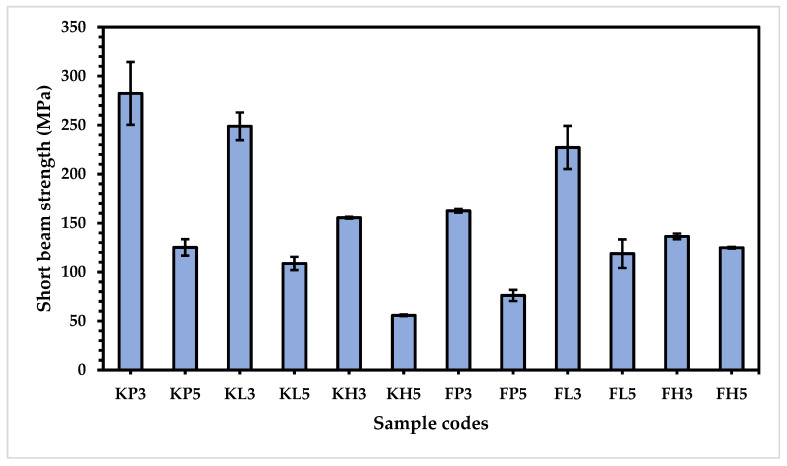
Short beam strength of composite samples.

After testing, bar charts were plotted to better understand the behavior of all samples in a convenient way, as shown in Figure 11. These results reveal that all types of five-layered composites have lower short beam strength than the three-layered composites of the same type. As the short beam test is used to assess the interlaminar shear strength of composites, when the number of layers increases in composites, the flexural property of the composite decreases, resulting in hardness of the composite samples. Thus, sudden and early destruction occurs due to applied loading, which causes the lower short beam strength of the five-layered composites.

The Kevlar-based composites offer a higher short beam strength than the flax-based composites for the three-layered samples; however, for the five-layered samples the flax-based composites FL3 (227.18 MPa) and FH3 (136.37 MPa) show higher short bending strength than the Kevlar-based samples KL3 (248.77 MPa) and KL3 (155.48 MPa) thanks to soft and elastic nature of flax, low melting polyester, and high-density polyethylene [41].

The short beam strength of the Kevlar-based composites with various polymer fibers demonstrates that the KP samples exhibit the highest short beam strength (282.32 and 125.09 MPa) as compared with KL (248.77 and 108.73 MPa), followed by KH (155.48 and 55.78 MPa) for three-layered and five-layered composites, respectively, because of spherulites present in the structure of polypropylene, which is highly oriented in the direction of the force. The deformation of this spherulite is elastic, and little or no disruption of the structure occurs. In addition, the spherulites are converted into microfibrils, resulting in the high short beam strength of the KP composites. On the other hand, the KH composites have the lowest short beam strength because of HDPE; when loading is applied, friction with HDPE is created, resulting in the generation of heat, which causes melting of the HDPE and interlaminar shear destruction of the composite.

The Flax-based composites show interesting behavior with respect to short beam strength. The FL composites hold the highest strength (227.18 and 118.75 MPa) compared to FP (162.59 and 76.11 MPa), followed by FH (136.37 and 124.74 MPa), for three-layered and five-layered composites, respectively [42]. The high short beam strength of FL can be attributed to the smaller branches in the long polymer chains of polypropylene, which make for strong interaction with the hemicellulose bonding of flax structure via ß1,4 linkage [43]. These results are supported by the fiber adhesion strength, as shown in Table 5.

### 3.4. Impact Properties

**Figure 12:**
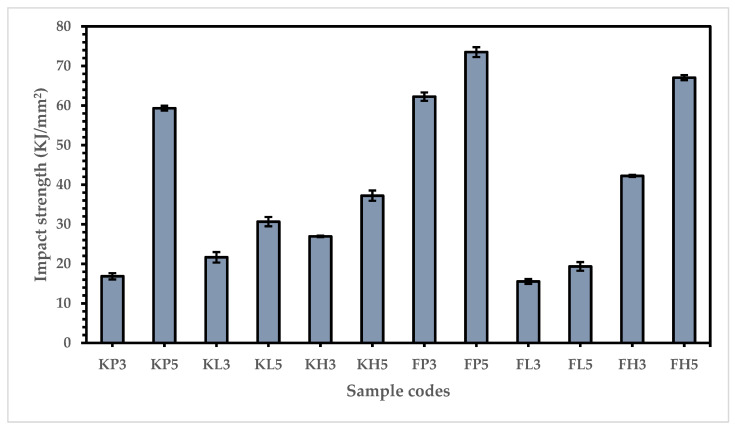
Impact strength of composite samples.

To ensure the safety of batsmen in case of the pads being hit by the fast speed of the ball or the batsman falling, it is necessary to understand the sudden effect/force. Therefore, the impact strength of all the composites was investigated, and the results are presented in Figure 12.

These results explain that all types of five-layered composites have higher impact strength than three-layered composites of the same types. This is due to the elastic behavior of the synthetic fibers available in the composites. They have more resilience, and can bear the effect of sudden force through elongation. Thus, five-layered composites exhibit higher impact strength than three-layered composites [44].

Most of the Flax-based composites offer a higher impact strength than the corresponding Kevlar-based composites because of their soft, elastic, and extremely long crystalline polymer system. This forms more hydrogen bonds, causing higher resistance to sudden loads and resulting in higher impact strength [45]. When comparing all flax-based composites with each other, the FP samples show the highest impact strength (62.24 KJ/mm^2^ and 73.51 KJ/mm^2^) compared to FH (42.21 KJ/mm^2^ and 67.05 KJ/mm^2^), followed by FL (15.54 KJ/mm^2^ and 19.35 KJ/mm^2^), for three-layered and five-layered composites, respectively. This could be because of the combined effect of flax and PP. Due to their high elongation and extremely long polymers, which can form more hydrogen bonds with each other, these composites result in increased impact strength [46]. These results are supported by the fiber adhesion strength, as shown in Table 5.

The same trend can be observed in the Kevlar-based composites, where KP samples show higher impact strength (KP3 = 16.85 KJ/mm^2^ and KP5 = 59.32 KJ/mm^2^) than KH (KH3 = 26.93 KJ/mm^2^ and KH5 = 37.23 KJ/mm^2^), followed by KL (KL3 = 21.65 KJ/mm^2^ and KL5 = 30.66 KJ/mm^2^). These results can be attributed to polypropylene (PP), which has the highest elongation due to its linear, highly crystalline, and geometrically regular structure (isotactic) as compared to low melting polyester (LMPET) and high-density polyethylene (HDPE), as well as the strong hydrogen bonding and several repeated inter-chain linkages in the chemical structure of Kevlar [47]. Although the KP5 composite exhibits enormous impact strength (KP5 = 59.32 KJ/mm^2^), the FP5 composite has a higher impact strength than even KP5. This high strength of FP5 can be attributed to the shorter fiber length of flax; fibers are broken down to their ultimate fiber cells via mechanical or chemical means, which can make for strong intermolecular bonding with the highly oriented and highly anisotropic microfibrils of polypropylene, resulting in the highest resistance against sudden load/force and triggers to enhance the impact strength of FP5, as proven by the results presented in Figure 12 [48].

### 3.5. Microscopic Images and Morphological Structure Analysis

**Figure 13:**
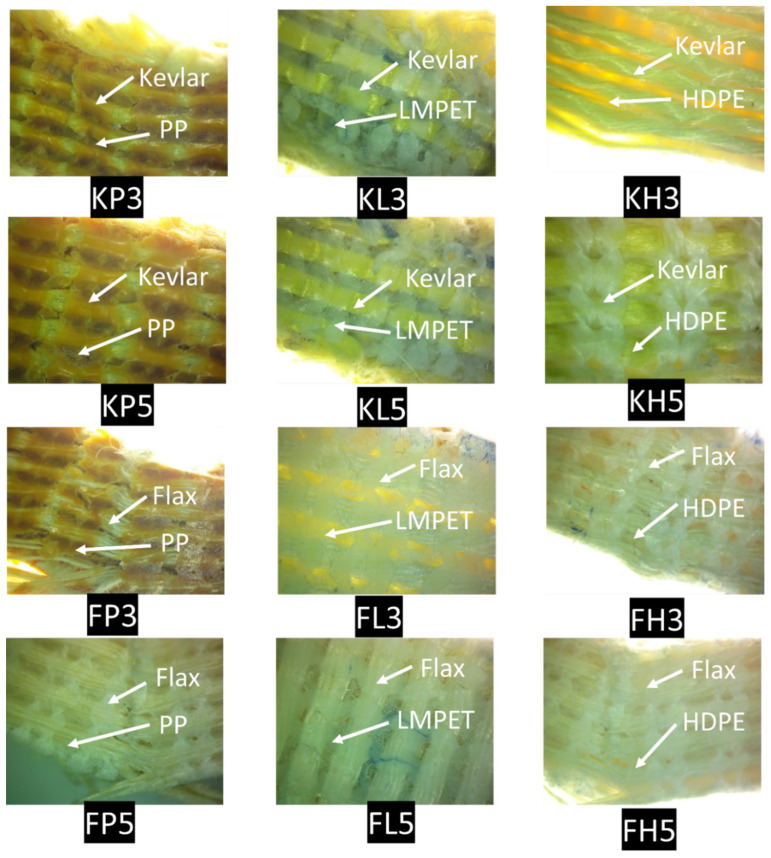
Light microscopic images of all composite samples.

The light microscopic images of all composite samples shown in Figure 13 reveal the clear and well-organized layers of flax and Kevlar fibers, which are strongly embedded with the matrix (PP, LMPET, and HDPE). Analysis reveals the proper bonding and adhesion of both Kevlar and flax fabrics with the thermoplastic yarn/matrix. In addition, it shows that the spacing between the layers of each composite are almost the same with respect to each other, resulting in efficient and careful designing of the composites. It can be seen from the fractured samples that a brittle fracture occurred due to fiber breakage.

### 3.6. Statistical Analysis

First, we defined the factors and their effect levels on impact, compression, tensile, and short beam strength. Then, experiments were carried out according to runs determined by orthogonal arrays in order to obtain the results. A decision matrix was created with the signal to noise ratio (S/N), as shown in Table 6. S/N ratios are computed to signify the quality characteristics of the responses. To obtain unit-free data sequences, normalized S/N ratios were used on each quality characteristic for a response, as shown in Table 7. Then, the TOPSIS method was employed to obtain the closeness to the ideal solution, the results were ranked, as shown in Table 8 [49].

Analysis of variance (ANOVA) was performed to identify the significant factors at α = 0.10 [50], with the results reported in Table 9.

The results show that effect of the number of layers (C) and inlay yarn (A) are more important factors with higher contributions. The ranking of the experimental runs based on their TOPSIS provides the optimal sample. The ranking is performed on the basis of major attributes, i.e., impact strength. On this basis, sample FP5 ranks first and is judged as the most ideal sample.

**Figure 14:**
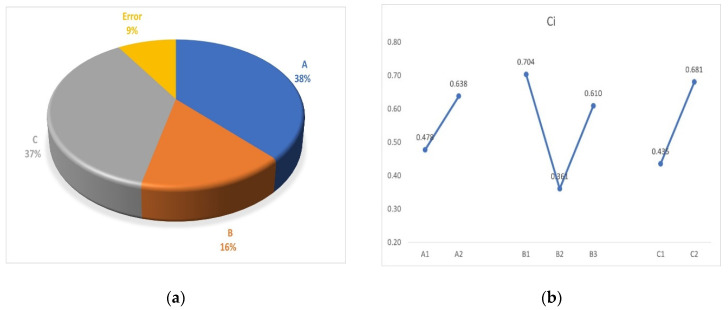
Percentage (%) contribution of each factor: (**a**) pie chart and (**b**) main effect.

The percentage contribution and main effects of the different factors are shown in Figure 14a,b.

When analyzed through the main effect plot, it can be concluded that the reinforcing material and number of layers have significant effects when determining the impact performance. The effect of the thermoplastic/knitting yarn is higher for PP, decreases for LMP, and then increases again for HDPE.

### 3.7. Confirmatory Test for the Most Ideal Sample

**Figure 15:**
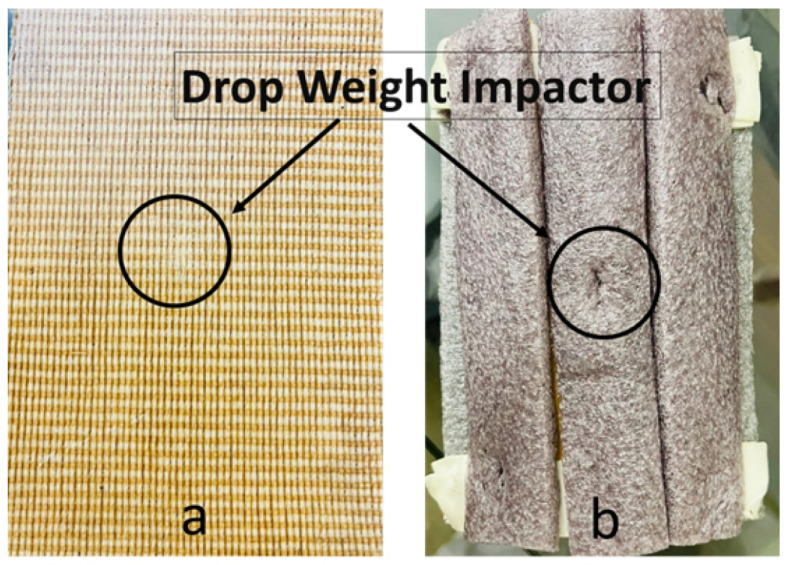
Samples used for drop weight impact test: (**a**) FP5 and (**b**) CP.

The most ideal sample, FP5, was tested using the drop weight impact tester, with the results shown in Figure 15. Furthermore, the drop weight impact performance of the five-layered flax/Polypropylene (FP5) composite was compared with aa commercial cricket pad (CP) from the market.

**Figure 16:**
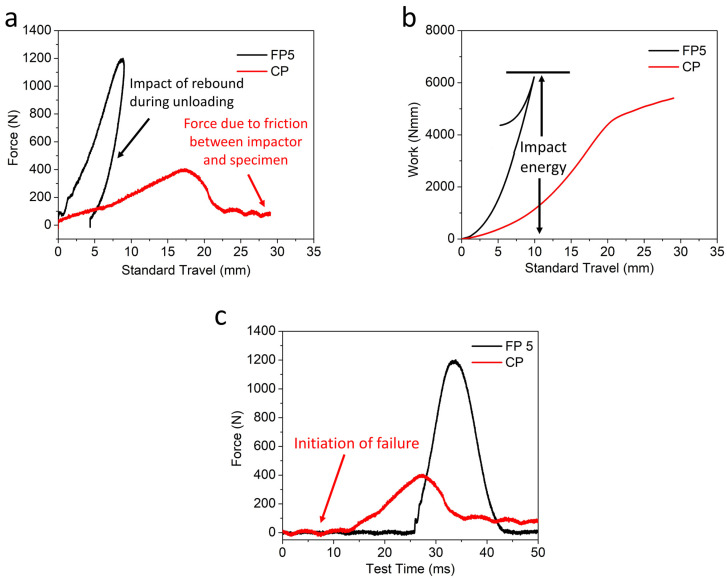
(**a**) Force standard travel curve, (**b**) work standard travel curve, and (**c**) force test time response of FP5 composite CP (cricket pad).

The average curve was plotted for better understanding, and is shown in Figure 16. (a) Force standard travel curve, (b) work standard travel curve, and (c) force test time response of FP5 composite CP (cricket pad).

The results reveal that the standard force travel curve exhibits a closed pattern; the area under the closed loop refers to the energy absorbed during the impact, as shown in Figure 16a. Further, the standard work travel curves are shown in Figure 16b.

As expected, the FP5 composite absorbs a much higher amount of impact energy and reaches its peak energy level at a lower deformation than the market CP (Cricket Pad) [51]. This is mainly due to the higher modulus, tenacity, and work to rupture of the FP5 composite compared to the CP sample [52]. This may be due to the interlaced five-layered structure of the FP composite, which provides more resistance during an impact failure than the CP sample. In addition, the CP sample breaks at much lower loads compared to the FP5 composite. At the initial portion of the curve of the CP sample, a sudden drop in load can be observed. This represents the initiation of matrix cracking and delamination [53], indicating the transition of the specimen from an intact state to a damaged state. The loading and unloading cycles are almost identical in the case of FP5, while they are not identical in the case of the sample CP, as shown in Figure 16c. This is because of the significant change in the strike velocity during impact testing. The above observations confirm the much better performance of the FP5 composite sample compared to commercial cricket pads from the market.

## 4. Conclusions

The sport of cricket requires greater impact performance on the part of personal protective equipment (PPE). Growing ecological, economic, and environmental awareness has driven efforts for the development of new materials and exploration of new applications of existing materials for various engineering applications. In the present study, the prime focus was to investigate different materials with good impact properties for use in cricket pads. As cricket balls are delivered at high velocities, they pose a threat to the safety of batsmen. The statistical techniques of the TOPSIS multi-response optimization method and ANOVA were employed to determine the performance of the samples we developed. A Flax-based five-layered polypropylene thermoplastic composite showed the best impact properties. This study provides evidence on the efficacy of the multi-response optimization methodology in determining the impact performance of flexible thermoplastic composites intended for use in sports equipment. Our results show that the proposed methodology is effective in determining the most ideal material for cricket pads. The most influential factors in determining the impact performance of knitted multilayered flexible composites are the number of layers and type of inlay yarn. Flax fiber is an attractive option with regard to its environmental friendliness. Thus, it can improve the impact performance of cricket pads on the one hand and utilize a renewable natural resource on the other. The developed FP5 sample shows the most ideal impact performance, and is far superior to the commercial sample from the market. Flax-based flexible composites show superior impact energy absorption, and could find applications in recreational activities other than cricket pad. Further research into the best materials for use in other forms of protective equipment is needed. Practical evaluation of the developed samples in real-world situations will be conducted in the future.

## Figures and Tables

**Table 1:** Properties of raw materials.

Filament Yarn Type	Molecular Weight	Linear Density (Denier)	Density (g/cm^3^)	Elongation (%)	Tensile Strength (MPa)	Melting Point (°C)
Flax	542.6	6000	1.44	2.96	1321	Not Available
Kevlar	274.27	6000	1.45	3.55	3082	555
High Density Polyethylene (HDPE)	177,450	600	0.98	2.75	1385	148
Polypropylene (PP)	354.6	600	0.88	60.85	274	155
Low Melting Polyester (LMP)	15,572	600	1.26	2.12	68	170

**Table 2:** Experimental factors and their levels.

Factors	Levels		
	1	2	3
**Inlay Yarn (A)**	Para Aramid (Kevlar)	Flax	-------
**Knitting Yarn (B)**	Polypropylene (PP)	Low Melting Polyester (LMP)	High Density Polyethylene (HDPE)
**Layers for composite (C)**	3	5	-

**Table 3:** Factors and their levels.

Coded Factors			Sample Code	Actual Factors		
A	B	C		A: Inlay Yarn	B: Knitting Yarn	C: Layers of Composite
1	1	1	KP3	Kevlar	Polypropylene	3
1	1	2	KP5	Kevlar	Polypropylene	5
1	2	1	KL3	Kevlar	Low Melting Polyester	3
1	2	2	KL5	Kevlar	Low Melting Polyester	5
1	3	1	KH3	Kevlar	High-Density Polyethylene	3
1	3	2	KH5	Kevlar	High-Density Polyethylene	5
2	1	1	FP3	Flax	Polypropylene	3
2	1	2	FP5	Flax	Polypropylene	5
2	2	1	FL3	Flax	Low Melting Polyester	3
2	2	2	FL5	Flax	Low Melting Polyester	5
2	3	1	FH3	Flax	High-Density Polyethylene	3
2	3	2	FH5	Flax	High-Density Polyethylene	5

**Table 4:** Physical parameters of knitted specimens.

Knitted Specimen	Knitting Yarn Stitch Length (mm)	Courses Density (cm^−1^)	Wales Density (cm^−1^)	Stitch Density (cm^−2^)
Kevlar/PP (KP)	0.6	5.91	2.75	16.24
Kevlar/LMP (KL)	0.6	5.51	2.75	15.16
Kevlar/HDPE (KH)	0.6	5.91	2.75	16.24
Flax/PP (FP)	0.6	5.51	2.75	15.16
Flax/LMP (FL)	0.6	5.51	2.75	15.16
Flax/HDPE (FH)	0.6	7.09	2.75	19.49

**Table 5:** Samples and their response details.

Sample Code	Responses				
	Fiber Pull-Out Strength (MPa)	Y1: Tensile Modulus (GPa)	Y2: Bending Modulus (GPa)	Y3: Short Beam Strength (MPa)	Y4: Impact Strength (KJ/mm^2^)
KP3	2.14 ± 0.11	26.77 ± 1.12	2.20 ± 0.07	282.32 ± 15.54	16.85 ± 1.47
KP5	2.14 ± 0.11	31.31 ± 1.45	2.90 ± 0.05	125.09 ± 11.42	59.32 ± 3.12
KL3	2.16 ± 0.08	28.27 ± 1.44	0.06 ± 0.01	248.77 ± 20.12	21.65 ± 1.75
KL5	2.16 ± 0.08	32.36 ± 1.17	0.56 ± 0.01	108.73 ± 17.24	30.66 ± 2.96
KH3	1.98 ± 0.04	22.65 ± 1.15	0.04 ± 0.01	155.48 ± 14.53	26.93 ± 4.44
KH5	1.98 ± 0.04	26.27 ± 1.14	2.34 ± 0.09	55.78 ± 07.51	37.23 ± 2.45
FP3	2.45 ± 0.12	7.67 ± 0.56	0.12 ± 0.01	162.59 ± 21.54	62.24 ± 5.13
FP5	2.45 ± 0.12	35.42 ± 1.18	6.04 ± 0.75	286.62 ± 22.21	73.51 ± 9.17
FL3	0.75 ± 0.12	6.21 ± 0.32	0.90 ± 0.11	227.91 ± 20.43	15.54 ± 0.92
FL5	0.75 ± 0.12	7.45 ± 0.23	1.68 ± 0.27	118.74 ± 18.11	19.35 ± 1.74
FH3	0.96 ± 0.08	9.08 ± 0.08	5.54 ± 0.07	136.37 ± 17.47	42.21 ± 2.15
FH5	0.96 ± 0.08	9.00 ± 0.10	6.69 ± 0.47	124.74 ± 12.31	67.06 ± 4.75

**Table 6:** Signal to noise (S/N) ratio and normalized S/N ratio.

Sample Code	S/N Ratio				Normalized S/N Ratio			
	Y1: Tensile Modulus (GPa)	Y2: Bending Modulus (GPa)	Y3: Short Beam Strength (MPa)	Y4: Impact Strength (KJ/mm^2^)	Y1: Tensile Modulus (GPa)	Y2: Bending Modulus (GPa)	Y3: Short Beam Strength (MPa)	Y4: Impact Strength (KJ/mm^2^)
KP3	28.481	6.760	48.903	24.512	0.839	0.778	0.991	0.051
KP5	29.857	9.201	41.904	35.463	0.930	0.833	0.495	0.862
KL3	29.013	−24.938	47.887	26.675	0.874	0.056	0.919	0.212
KL5	30.170	−5.065	40.695	29.718	0.950	0.508	0.409	0.437
KH3	26.991	−27.386	43.833	28.603	0.741	0.000	0.632	0.354
KH5	28.302	7.316	34.928	31.407	0.827	0.791	0.000	0.562
FP3	17.632	−18.404	44.221	35.879	0.125	0.205	0.659	0.893
FP5	30.924	15.604	49.023	37.324	1.000	0.979	1.000	1.000
FL3	15.736	−0.933	47.065	23.816	0.000	0.603	0.861	0.000
FL5	17.421	4.495	41.362	25.704	0.111	0.726	0.456	0.140
FH3	19.157	14.849	42.691	32.508	0.225	0.962	0.551	0.643
FH5	19.085	16.511	41.920	36.528	0.221	1.000	0.496	0.941

**Table 7:** Weighted and normalized decision matrix.

Sample Code	Weighted and Normalized Decision Matrix			
	Y1: Tensile Modulus (MPa)	Y2: Bending Modulus (GPa)	Y3: Short Beam Strength (MPa)	Y4: Impact Strength (KJ/mm^2^)
KP3	0.074	0.248	0.139	0.023
KP5	0.082	0.266	0.069	0.391
KL3	0.077	0.018	0.129	0.096
KL5	0.083	0.162	0.057	0.198
KH3	0.065	0.000	0.088	0.161
KH5	0.073	0.253	0.000	0.255
FP3	0.011	0.065	0.092	0.405
FP5	0.088	0.313	0.140	0.453
FL3	0.000	0.192	0.120	0.000
FL5	0.010	0.232	0.064	0.063
FH3	0.020	0.307	0.077	0.292
FH5	0.019	0.319	0.069	0.426

**Table 8:** Positive ideal (Si+), Negative ideal (Si−), and Closest ideal (Ci).

Sample Code	Si+	Si−	Ci	Rank
KP3	0.436	0.295	0.403	8
KP5	0.108	0.485	0.817	3
KL3	0.468	0.179	0.276	12
KL5	0.311	0.275	0.470	7
KH3	0.437	0.194	0.308	11
KH5	0.252	0.366	0.592	6
FP3	0.274	0.420	0.605	5
FP5	0.007	0.575	0.989	1
FL3	0.479	0.227	0.322	10
FL5	0.414	0.249	0.376	9
FH3	0.187	0.431	0.698	4
FH5	0.102	0.538	0.841	2

**Table 9:** Results of ANOVA.

Factors	Degrees of Freedom (DF)	Adjusted Sum of Squares (SS)	Adjusted Mean of Squares (MS)	F-Value	*p*-Value
A	1	0.127	0.127	4.260	0.078
B	2	0.105	0.052	1.760	0.241
C	1	0.125	0.125	4.200	0.080
Error	7	0.209	0.030		
Total	11	0.566			

## Data Availability

Data sharing not available.
